# P-1971. Risk Profiling of Severe Cutaneous Adverse Reactions (SCARs) to Antibiotics: A Disproportionality Analysis Using the FAERS Database

**DOI:** 10.1093/ofid/ofaf695.2138

**Published:** 2026-01-11

**Authors:** Alvin Sunny, Linta Susan Kuriakose, Albin C Sebastian

**Affiliations:** Square Hospital, West Panthapath, Dhaka, Bangladesh; Square Hospital, West Panthapath, Dhaka, Bangladesh; Square Hospital, West Panthapath, Dhaka, Bangladesh

## Abstract

**Background:**

Severe Cutaneous Adverse Reactions (SCARs), including Stevens-Johnson syndrome (SJS), toxic epidermal necrolysis (TEN), drug reaction with eosinophilia and systemic symptoms (DRESS), and acute generalized exanthematous pustulosis (AGEP), are rare but potentially fatal outcomes of antibiotic exposure. Early identification of drug-specific risk is essential to guide safer prescribing. This study aimed to evaluate and compare SCAR signals associated with commonly used antibiotics using the U.S. FDA Adverse Event Reporting System (FAERS).

**Figure:**
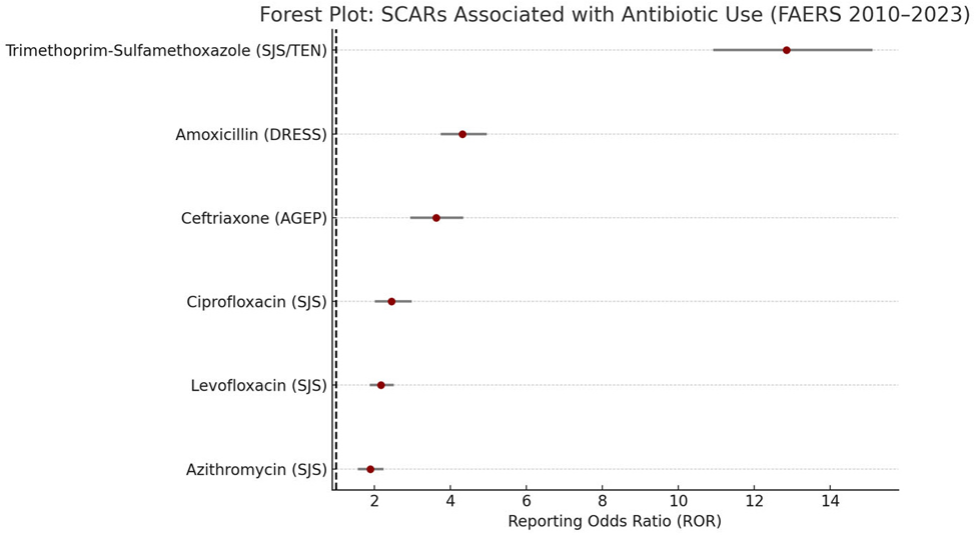
Forest Plot: SCARs Associated with Antibiotic Use (FAERS 2010–2023) This forest plot presents the reporting odds ratios (RORs) with 95% confidence intervals for severe cutaneous adverse reactions (SCARs) related to commonly used antibiotics. Trimethoprim-sulfamethoxazole showed the strongest signal for SJS/TEN, followed by significant signals for amoxicillin (DRESS), ceftriaxone (AGEP), and fluoroquinolones (SJS). All antibiotics listed demonstrated statistically significant associations, underscoring the importance of hypersensitivity risk profiling in clinical practice.

**Methods:**

A retrospective pharmacovigilance analysis was conducted using FAERS data from January 2010 to December 2023. Reports mentioning SCAR-related MedDRA Preferred Terms were extracted. Antibiotics assessed included beta-lactams, macrolides, fluoroquinolones, sulfonamides, and tetracyclines. Signal detection was performed using disproportionality analysis—calculating Reporting Odds Ratios (ROR) and 95% confidence intervals (CI). A signal was considered positive if the ROR lower CI exceeded 1 with ≥3 reports.

**Results:**

A total of 5,294 SCAR-related reports were linked to antibiotic exposure. Sulfonamides, particularly trimethoprim-sulfamethoxazole, showed the strongest signal for SJS/TEN (ROR: 12.84, 95% CI: 10.92–15.11). Beta-lactams—especially amoxicillin and ceftriaxone—were significantly associated with DRESS and AGEP (ROR: 4.31 and 3.62, respectively). Fluoroquinolones demonstrated moderate signals for SJS, while macrolides showed weaker but present associations. The majority of severe cases required hospitalization, and 8.3% resulted in reported death.

**Conclusion:**

This FAERS-based analysis confirms that sulfonamides and certain beta-lactams carry the highest SCAR risk among antibiotics. The findings support heightened clinical vigilance and risk stratification, especially in patients with known drug allergies or genetic predispositions. Continuous pharmacovigilance is crucial for identifying high-risk agents and improving patient safety in antibiotic prescribing.

**Disclosures:**

All Authors: No reported disclosures

